# Evaluating emotional competencies in health professionals in a city in Colombia

**DOI:** 10.1192/j.eurpsy.2024.1231

**Published:** 2024-08-27

**Authors:** E. P. Ruiz Gonzalez, M. N. Muñoz Argel, S. Garcia, A. Sierra

**Affiliations:** ^1^ Universidad Pontificia Bolivariana; ^2^Fundacion Saecivi@gmail.com, Montería, Colombia

## Abstract

**Introduction:**

Emotional competencies, according to Bisquerra Alzina & Escoda (2007), refer to “knowledge, skills and attitudes necessary to understand, express and appropriately regulate emotional phenomena” (p. 22) in the management of emotions with oneself and with the other.

**Objectives:**

Measure the emotional competencies Empathy, Emotional Expression and Emotional Regulation in health professionals in healthcare centers.

**Methods:**

Quantitative descriptive. The Inventory of Emotional Competencies for Adults (Mikulic, Crespi, Radusky, 2015) was applied to 30 participants (doctor, psychologist, nurse, dentist).

**Results:**

The grouped measurements show skills at a medium and high level.

**Table 1.** Measurement of empathy capacity, emotional regulation capacity and emotional expression capacity

		Frequency	Porcent
Empathy	Medium	27	90,0
	High	3	10,0
Emotional Regulation	Medium	15	50,0
	High	15	50,0
Emotional Expression	Medium	26	86,7
	High	4	13,3
	Total	30	100,0

The emotional reaction of congruence with the emotional state of the other, empathy, shows a medium level (Table 1), a result consistent with the study by Ruiz González (2019), in the Colombian population, where a medium level of empathy is observed in doctors.

In the strategy for management, support, increase and suppression of the current affective state to self-soothe and find a state of relaxation, it is at an average value between medium and high (table 1.)

In the ability to start and maintain conversations, express one’s own thoughts and feelings clearly, both in verbal and non-verbal communication, and demonstrate to others that they have been well understood, the level is mostly medium (table 1.)

**Conclusions:**

The levels of emotional competencies evaluated are mostly in the middle in the assessment by dimensions, empathy registered a lower level in contrast to other dimensions.Taking into consideration professional practice, response to organic and mental human vulnerability, it is a field for promoting the well-being of the health professional.

**Disclosure of Interest:**

None Declared

